# Impact of Race and Socioeconomics Disparities on Survival in Young-Onset Colorectal Adenocarcinoma—A SEER Registry Analysis

**DOI:** 10.3390/cancers13133262

**Published:** 2021-06-29

**Authors:** Mark M. Aloysius, Hemant Goyal, Niraj J. Shah, Kumar Pallav, Nimy John, Mahesh Gajendran, Abhilash Perisetti, Benjamin Tharian

**Affiliations:** 1 Department of Internal Medicine, The Wright Center for Graduate Medical Education, 501 S. Washington Avenue, Scranton, PA 18505, USA; madhoka@thewrightcenter.org; 2 Division of Digestive Diseases, University of Mississippi Medical Center, 2500 North State Street, Jackson, MS 39216, USA; jnshah@umc.edu; 3 Division of Gastroenterology and Hepatology, University of Texas Medical Branch, Galveston, TX 77555, USA; kupallav@utmb.edu; 4 Department of Gastroenterology and Hepatology, The University of Arkansas for Medical Sciences, Little Rock, AR 72205, USA; njohn@uams.edu (N.J.); aperisetti@uams.edu (A.P.); btharian@uams.edu (B.T.); 5 Department of Internal Medicine, Texas Tech University Health Science Center El Paso, 2000B Transmountain Road, El Paso, TX 79911, USA; Mahesh.gajendran@ttuhsc.edu

**Keywords:** colon cancer, colorectal cancer, poverty, socioeconomic determinants of health, survival, race

## Abstract

**Simple Summary:**

The results of this study show the effects of socio-economic determinants, such as higher income levels, high school education, private insurance, and married marital status, have favorable survival in patients with young-onset colorectal cancer (YoCRC). Moreover, most of the positive social factors are often interrelated. The inclusion of these factors could further prognosticate and help with healthcare resource allocation for successful interventions through public health measures. Colorectal cancer awareness, knowledge, and even utilization of medical services would differ with the education and health literacy.

**Abstract:**

Introduction: We aimed to assess the impact of socio-economic determinants of health (SEDH) on survival disparities within and between the ethnic groups of young-onset (<50 years age) colorectal adenocarcinoma patients. Patients and Methods: Surveillance, epidemiology, and end results (SEER) registry was used to identify colorectal adenocarcinoma patients aged between 25–49 years from 2012 and 2016. Survival analysis was performed using the Kaplan–Meir method. Cox proportional hazards model was used to determine the hazard effect of SEDH. American community survey (ACS) data 2012–2016 were used to analyze the impact of high school education, immigration status, poverty, household income, employment, marital status, and insurance type. Results: A total of 17,145 young-onset colorectal adenocarcinoma patients were studied. Hispanic (H) = 2874, Non-Hispanic American Indian/Alaskan Native (NHAIAN) = 164, Non-Hispanic Asian Pacific Islander (NHAPI) = 1676, Non-Hispanic black (NHB) = 2305, Non-Hispanic white (NHW) = 10,126. Overall cancer-specific survival was, at 5 years, 69 m. NHB (65.58 m) and NHAIAN (65.67 m) experienced worse survival compared with NHW (70.11 m), NHAPI (68.7), and H (68.31). High school education conferred improved cancer-specific survival significantly with NHAPI, NHB, and NHW but not with H and NHAIAN. Poverty lowered and high school education improved cancer-specific survival (CSS) in NHB, NHW, and NHAPI. Unemployment was associated with lowered CSS in H and NAPI. Lower income below the median negatively impacted survival among H, NHAPI NHB, and NHW. Recent immigration within the last 12 months lowered CSS survival in NHW. Commercial health insurance compared with government insurance conferred improved CSS in all groups. Conclusions: Survival disparities were found among all races with young-onset colorectal adenocarcinoma. The pattern of SEDH influencing survival was unique to each race. Overall higher income levels, high school education, private insurance, and marital status appeared to be independent factors conferring favorable survival found on multivariate analysis.

## 1. Introduction

Colorectal cancer (CRC) is the third most commonly diagnosed cancer and the third leading cause of cancer deaths in both men and women in the United States, with an estimated 147,950 people diagnosed and 53,200 deaths in 2020 [1]. From 1975 to 2017, the overall CRC incidence and mortality trend have decreased by more than 30% and 50%, respectively [2]. However, this trend was mainly seen in screen-eligible populations above 50 years of age [3]. In marked contrast, the incidence of young-onset colorectal cancer (YoCRC), traditionally defined by diagnosis under age 50, has increased by 26% from 8.5 per 100,000 in 1992 to 10.7 per 100,000 in 2013 [2,4,5]. Further, it has been estimated that by the year 2030, 10% of CRC would likely develop in people under the age of 50 years [6,7]. Among the strategies agreed upon for improving cancer survival in CRC, health awareness and education of low socioeconomic groups have been suggested [8]. Equitable distribution of healthcare is a desirable trait of any developed society. In the U.S, glaring and wide-ranging disparities exist in colorectal cancer survival [9]. The interpretation of this association needs to be cautiously performed as affluence may provide health care affordability but by itself may not guarantee better health outcomes unless it is also associated with better health literacy. There is a growing body of literature that ethnicity is a major determinant in colorectal cancer treatment outcomes and several other factors [10,11]. Given the complexity of multiple factors driving health care disparities, any attempt to mitigate and remediate them is doomed to fail without a pivotal understanding of the interplay between various racial and socioeconomic determinants (SEDH) impacting availability and access to care. With the rising incidence of YoCRC, there are compelling recommendations to lower the age of screening to 45 [12,13].

The influence of these factors on mortality within each racial/ethnic group has not been studied in YoCRC. Resource allocation for successful interventions through public health measures requires a better understanding of the impact of SEDH on YoCRC survival within each vulnerable racial/ethnic group of a diverse population. Outcome disparities exist for CRC between African Americans and Caucasians and can be attributed to the socioeconomic status [14,15]. Another key determinant is high school education, which has been shown to increase the odds of CRC screening by 2.47 times when compared with those without high school education [16,17]. Hence, our study aimed to determine the effects of multiple SEDH on survival between different racial/ethnic groups with YoCRC.

## 2. Materials and Methods

### 2.1. Study Design

This is a retrospective cohort study of Surveillance Epidemiology and End Results Program-American Census Survey (SEER-ACS) data. Since it is a de-identified dataset, the study was exempted for a review/approval by the institutional review board of the Wright Center for Graduate Medical Education (IRB reference—1698777-1).

### 2.2. Data Source

The SEER database contains comprehensive patient outcome data, including mortality from 18 population-based cancer registries covering approximately a third of the U.S. population [18]. Data extraction was through a case listing session initiated through SEER*Stat software version 8.3.8 (^®^ NCI) run on the combined SEER 18 incidence registries to obtain demographic, tumor characteristics, and survival data on histologically proven (adenocarcinoma) YoCRC patients [19]. The ACS data integrated into the SEER database were used concomitantly and are updated every 4 years [20].

### 2.3. Patient Selection

We limited our analysis to include patients diagnosed during the years 2012–2016 to overlap with a single period of census survey. The rationale behind this is that SEDH (socioeconomic determinants of health) data accrued from ACS occurs in fixed time periods, and we used SEER data from 2012–2016 as the congruent matched dataset to ACS 2012–2016 for meaningful, accurate analysis within a SEDH unchanged fixed time frame. Another reason to choose the dataset from 2012–2016 was based on American Joint Committee on Cancer (AJCC) staging. SEER data prior to 2012 used a different AJCC stage edition VI with less delineation of sub-stages, whereas 2012–2016 used AJCC stage system VII, which has more substage differentiation. In this study, we defined YoCRC as cancers of the colon and rectum diagnosed in individuals 25 to 49 years old at the time of diagnosis. The race/ethnicity was classified as Hispanic (H), Non-Hispanic American Indian/Alaskan Native (NHAIAN), Non-Hispanic Asian Pacific Islander (NHAPI), Non-Hispanic black (NHB). We excluded patients with histopathologic subtypes other than adenocarcinoma (such as neuroendocrine, squamous cell, sarcoma etc.), patients with multiple primaries, un-stageable cancers (AJCC-TNM) and patients with incomplete survival data. Patients were followed up for a minimum duration of 5 years.

### 2.4. Socioeconomic Determinants of Health (SEDH)

An area-based measure of seven SEDH parameters available in SEER was used in concurrence with the survival data. [https://seer.cancer.gov/seerstat/variables/countyattribs/static.html#12-16. Accessed in 3 March 2021]. The SEDH data were assessed whether above or below the 50th centile [19]. The parameters defined by the county attribute table of the ACS-census bureau were marital status, employment status, poverty (as defined by ACS based on the U.S. census 2012–2016), immigration status (immigrated to the U.S. within the last 12 months), high school education (completed), insurance status (private vs. Medicaid), and household income (median income as defined by U.S. census 2012–2016) [19].

### 2.5. Statistical Analysis

The demographic and tumor characteristics stratified by race/ethnicity were compared by chi-square test for categorical variables and *t*-test for continuous variables. Median survival (months) was calculated using the Kaplan–Meier analysis. The 5-year survival was analyzed using Log-rank tests and Cox proportional hazards models. Survival time was determined from the date of diagnosis to the last date of follow-up or until the date of death that was cancer-specific as made available through SEER. Hazard ratios (H.R.s) and 95% C.I.s were estimated for univariate and multivariate analysis, using the Cox proportional hazard model. Cancer-specific survival was evaluated for all patients stratified by race/ethnicity and combined as univariate analysis for each SEDH. Cox proportional hazards assumption in the multivariate models were also tested by adding an interaction term with race and follow-up time to the final models. This interaction term was not significant for the overall model or for the models stratified multiple SEDH. All data were analyzed using SPSS v27 for Macintosh (^®^ IBM). As per the data user agreement with SEER, any cell value <11 was censored to prevent re-identification of the rare events.

## 3. Results

**Figure 1:**
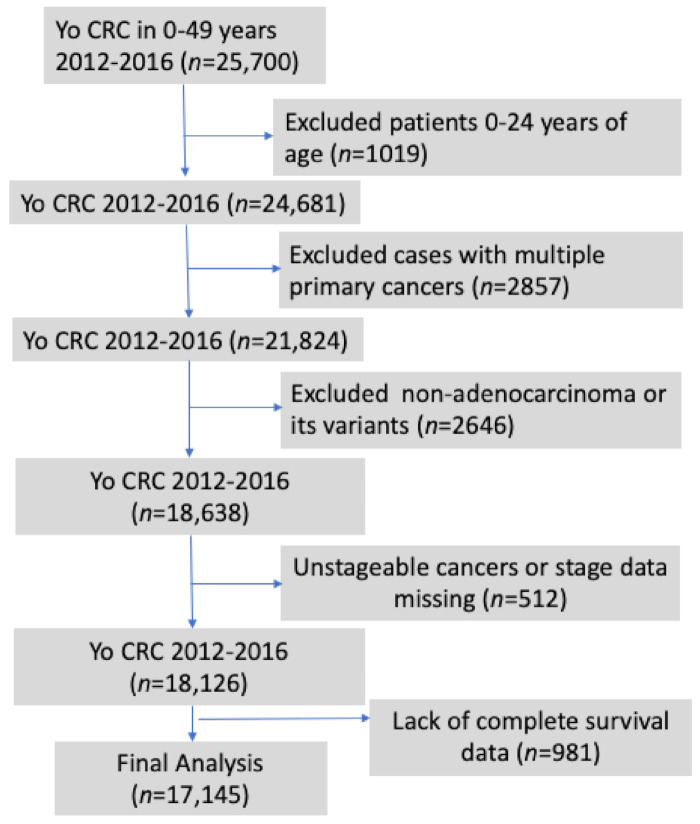
Systematic method used to extract patient information for the study from SEER database.

A total of 17,145 young-onset colorectal adenocarcinoma patients were studied (Figure 1). A summary of demographic and clinicopathologic characteristics of cancer patients included in the study is outlined in Table 1. The mean age of the cohort is 42.82 (+/−5.61 S.D.), with 51.9% males. In terms of race and origin, 59.1% were NHW, 16.8% were H, 13.4% were NHB, 9.8% were NHAPI, and 1% were NHAIAN.

**Figure 2:**
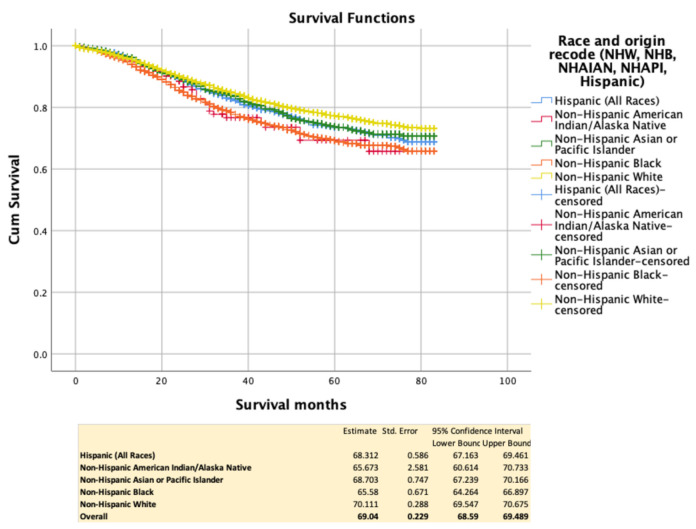
Kaplan–Meir cancer-specific survival between racial groups.

Overall and race-specific survival Overall cancer-specific survival was, at 5 years, 69 m. NHB (65.58 m) and NHAIAN (65.67 m) experienced worse survival when compared with NHW (70.11 m), NHAPI (68.7 m), and H (68.31 m). This is illustrated in Figure 2. A summary of race-specific survival controlled for each SEDH is summarized in Table 2.

### 3.1. Completion of High School Education (above vs. below 50th Centile)

**Figure 3:**
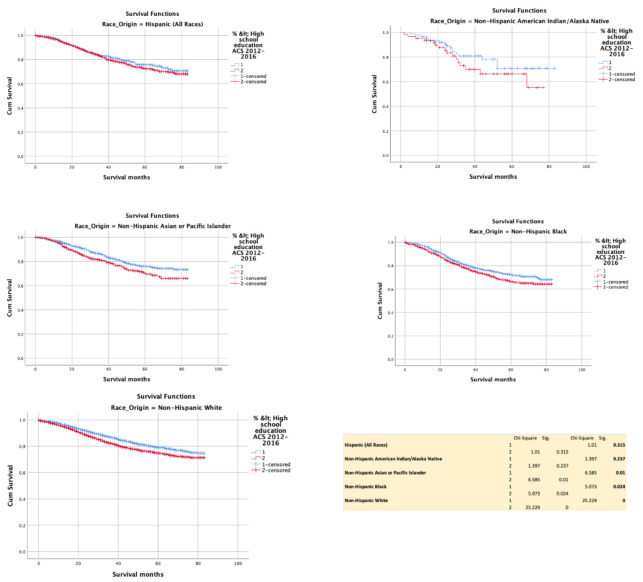
Kaplan–Meir survival curves showing the impact of high school education.

Completion of high school education was associated with improved cancer-specific survival significantly with NHAPI (*p* = 0.010), NHB (*p* = 0.024), and NHW (*p* < 0.0001). The effect size of improved HR was 0.795 (0.738–0.857). However, no difference was noted with H (*p* = 0.315) and NHAIAN (*p* = 0.237) (Figure 3).

### 3.2. Poverty (above vs. below 50th Centile)

**Figure 4:**
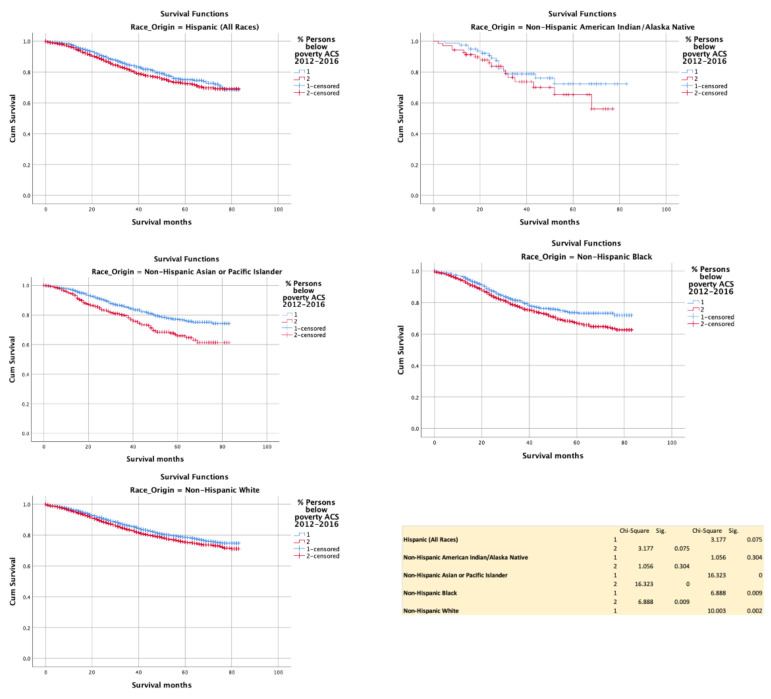
Kaplan–Meir survival curves showing the impact of poverty status.

Poverty adversely impacted cancer specific survival in NHB (*p* = 0.009), NHW (*p* = 0.315) and NHAPI (*p* = 0.002). The effect size of improved HR was 0.811 (0.753–0.875). However, no difference was noted with H (*p* = 0.075) and NHAIAN (*p* = 0.304) (Figure 4).

### 3.3. Employment Status (above vs. below 50th Centile) 

**Figure 5:**
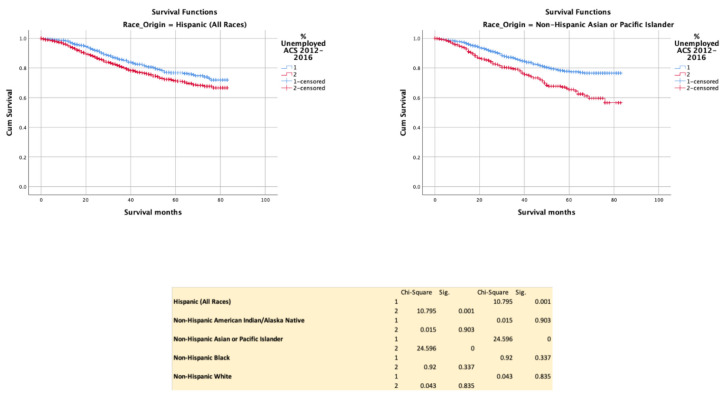
Kaplan–Meir survival curves showing the impact of employment status.

Unemployment negatively influenced survival in H (*p* = 0.001) and NHAPI (*p* < 0.0001), but not with NHAIN (*p* = 0.903), NHB (*p* = 0.337) or NHW (*p* = 0.835) (Figure 5).

### 3.4. Household Income with Reference to the Median (above vs. below 50th Centile

**Figure 6:**
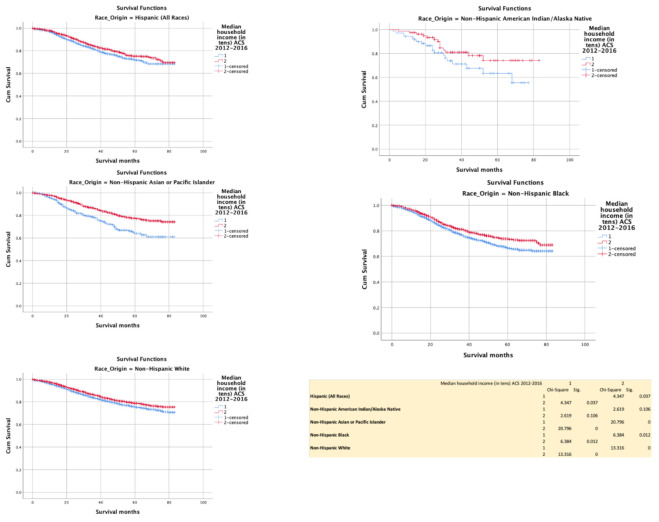
Kaplan–Meir survival curves showing the impact of household income.

Household income below median negatively impacted survival among NHAPI (*p* < 0.0001), NHB (*p* = 0.012), NHW (*p* < 0.0001), but not NHAIN (*p* = 0.106)) (Figure 6).

### 3.5. Marital (Married) Status (above vs. below 50th Centile) 

**Figure 7:**
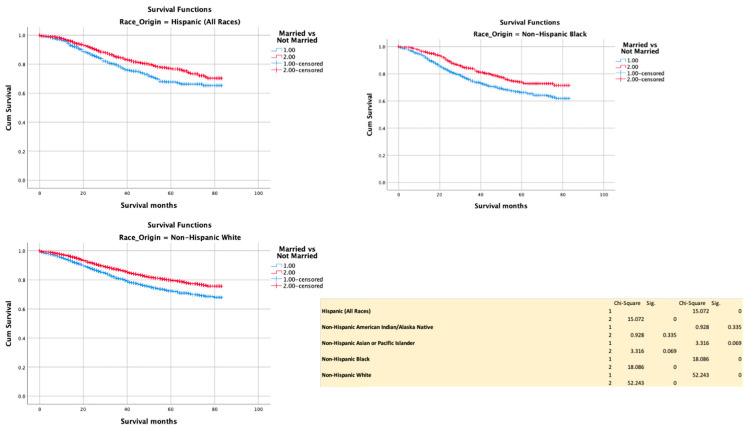
Kaplan–Meir survival curves showing the impact of marital status.

Being married conferred a survival advantage in H (*p* < 0.0001), NHB (*p* = < 0.0001) and NHW (*p* = 0.0001) but not in NHAIN (*p* = 0.335) or NHAPI (*p* = 0.069) (Figure 7).

### 3.6. Insurance (Commercial vs. Medicaid) 

**Figure 8:**
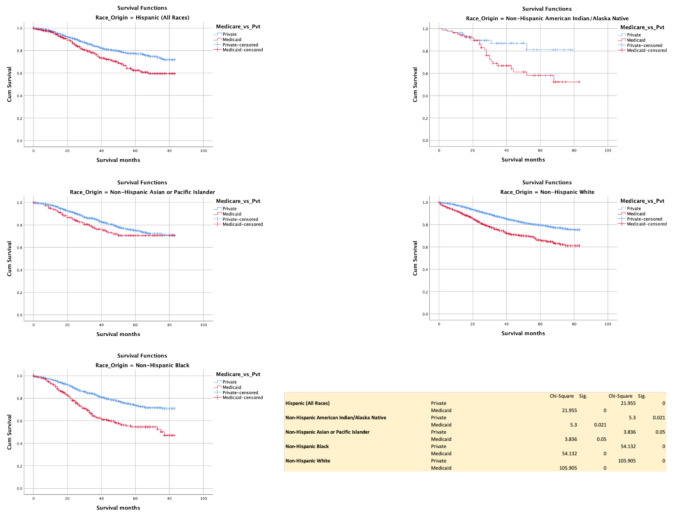
Kaplan–Meir survival curves showing the impact of insurance status.

Commercially insured patients had better survival compared with Medicaid in all races, H (*p* = < 0.0001), NHB (*p* < 0.0001), NHW (*p* < 0.0001), NHAIN (*p* = 0.021), and NHAPI (*p* = 0.05) (Figure 8).

### 3.7. Recent Immigration to the U.S. (above vs. below 50th Centile) 

**Figure 9:**
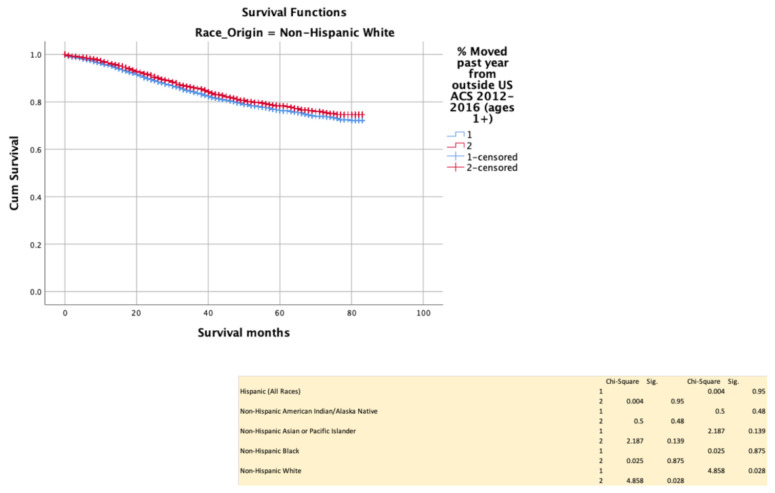
Kaplan–Meir survival curves showing the impact of immigration status.

Recent immigrant status conferred a survival disadvantage among NHW (*p* = 0.028), but not in other ethnic groups (Figure 9).

Overall Univariate and Multivariate model of survival controlling for SEDH interactions.

A result of the univariate and multivariate analyses performed is outlined in Table 3. Based on univariate analysis, each of the SEDH variables had a significant impact on the survival outcome. However, when all these factors were modeled in the multivariate analysis, the following factors were independent predictors of superior survival: poverty status, completion of high school education (HR 0.88, 95% CI 0.79–0.97), private insurance (HR 0.60, 95% CI 0.54–0.66), and married marital status (HR 1.31, 95% CI 1.20–1.42).

## 4. Discussion

Our analysis of outcomes for 17,145 individuals with YoCRC identified significantly worse 5-year survival for NHB among all races. The SEDH factors that predicted decreased survival include lower income levels, a lack of high school education, Medicare insurance, and unmarried status. Our analysis of the data obtained from the SEER cancer registries is unique, as we have for the first time focused and incorporated most of the socioeconomic determinants of health in determining cancer survival in YoCRC.

One of the key findings in our study of YoCRC patients is that the NHB race had worse 5-year survival rates when compared with other races. Based on previous studies, the reasons for these lower survival rates could be related to the lower rate of CRC screening, lower socioeconomic status, and less access to high caliber cancer treatment modalities, which could all result in missed lesions, late diagnosis, delay in the staging of the disease [21]. Previous studies on racial disparity in the survival of CRC, including YoCRC, have demonstrated poorer survival outcomes for NHB when compared with NHW [9,22,23]. Better awareness and screening guidelines and strategies suggested by multiple societies have led to an increase in CRC incidence across all racial groups. At 12.7 per 100,000 persons, the NHB individuals have the highest overall incidence of YoCRC [24,25]. Studies have indicated it is less likely that the NHB population will receive a follow-up colonoscopy, or even a high-quality colonoscopy, contributing to worse outcomes when compared with other ethnicities [26,27].

In our study, the household income below the median had a negative impact on the survival among NHAPI, NHB, and NHW, but not in NHAIN. In addition, our data indicated that the YoCRC patients in the poverty category had adversely affected the survival in NHB, NHAPI, and NHW, but not in H and NHAIAN. As per the 2018 U.S. census bureau, the median USA annual family income was higher in NHW (USD 70,642) when compared with NHB (USD 41,361). Household income in larger families may contribute to reduced affordability to seek access to health care and accompanying costs. As per the 2018 U.S. Census Bureau, an estimated 21% of NHB and 8% of NHW live under the poverty line [28]. It was demonstrated that employment and lower socioeconomic status positively correlate with exposure to CRC risk factors [29]. Lack of financial resources is a major barrier to CRC screening in adults over 50 years [30]. However, the average risk of YoCRC is currently not within screening guidelines, except for high-risk features, such as a strong family history or long-standing inflammatory bowel disease. It is quite possible that lack of affordability may be a significant factor in the underutilization of specialist gastroenterology services or other specialist cancer services within this population. Another possible reason could be that patients with low socio-economic status have poorly balanced diet, uncontrolled diabetes, and are at high risk of alcoholism and tobacco abuse, which are associated with inflammation and could be a trigger for abnormal immune response and CRC. Unfortunately SEER does not provide patient-specific information about these data. A SEER registry study by Scally et al. showed all CRC in adults over 18 years of age, spatial social polarization, quantified in relation to racialized economic segregation, increases the odds of late diagnosis of colorectal cancer for persons residing in the least compared with most privileged counties [31].

Married patients may have potentially greater financial resources and social support, especially if the spouse is employed, and are believed to have better CRC survival when compared with single or divorced patients. In our study, being married conferred a survival advantage in H, NHB, and NHW but not in NHAIN or NHAPI. In a comparative study from the SEER database, Wang et al. demonstrated marriage to be associated with lower mortality for married patients in the patients diagnosed with CRC after adjusting for age, race, cancer stage, and surgery [32]. In that study, married individuals were more likely to have an early stage of CRC diagnosis and therefore more likely to engage in CRC treatment when compared with single or divorced patients. In the CRC survivorship care guidelines issued by the American Cancer Society, there has been an increased emphasis on social support [33]. This is essentially required to help facilitate care decisions and accompany patients to appointments, chemotherapy, or hospital stay and for emotional and psychological support.

Our analysis revealed recent immigrant status to confer a survival disadvantage only amongst NHW, but not in other ethnic groups. As per the National Health Interview Survey from 2018, CRC screening was low amongst individuals with lesser than high school education (at 52%), uninsured (at 30%), and recent immigrants (<10 years, at 26%) [34]. For YoCRC adenocarcinomas, we report a better survival advantage with patients covered by private insurance over Medicaid. Myerson et al. demonstrated that universal affordability to health care over 65 years of age has a favorable impact on cancer detection and survival outcomes compared with no insurance [35]. A study from the national cancer database concluded that universal insurance coverage accounted for a 47% relative decrease in survival disparity in NHB compared with NHW with CRC [35,36]. However, no previous study has looked at health insurance and survival outcomes in YoCRC in the U.S. A study from Thailand by Surachai et al. showed that universal health coverage was associated with poor survival in colorectal cancer [37]. Similarly, we also note a survival advantage among patients with private insurance in the U.S., possibly related to better access to high-quality treatment centers and specialists when compared with Medicaid. This may perhaps indicate better access to state-of-the-art, cutting-edge treatments to those who have private insurances compared with Medicaid.

Despite a declining trend of overall CRC incidence and mortality in the U.S., NHB patients continue to experience a higher CRC mortality burden than NHW patients [38,39]. Mental-health risk and sociodemographic factors may also serve as barriers to CRC symptom screening among homeless black and white individuals, reflective of primary care underutilization [40].

In contrast to CRC over 50 years, YoCRC has not been traditionally detected through screening modalities but through symptomatic presentation to primary care, emergency rooms, or specialist services. Wu et al. demonstrated worse overall survival for YoCRC patients for NHB patients compared with NHW patients [22]. In our study, we also show cancer-specific survival being poor for NHB compared with NHWs. We hypothesize that the racial disparities in survival observed in our study are unlikely to be the effect of screening disparity but instead on prompt symptom recognition and utilization of health care. Hence, socioeconomic determinants such as health awareness, health literacy, affordability based on health insurance, immigration status and marital status, access to and utilization of health care services may heavily influence survival in YoCRC.

Our study has a few limitations. SEER cancer registry data do not include detailed information about chemotherapy regimens, the number of cycles, or radiotherapy treatments, making it impossible to determine which subjects received adjuvants chemotherapy or radiation therapy. Incomplete and underreported data in SEER is also a limitation; however, we excluded entries with incomplete data. Moreover, the SEER insurance variable does not subdivide those with private insurance (managed care, health maintenance organization, or preferred provider organization), and the differences between these subsets could not be analyzed. SEER registries also lack data on age or ethnicity matched controls, co-morbidities, environmental history, family history, tumor genotype (microsatellite instability), which are linked with treatment and outcomes for YoCRC. Despite these limitations, our study has important implications for resource allocation for eliminating barriers determining unfavorable outcomes for YoCRC. We also analyzed the socioeconomic status for different age groups and there was no statistically significant difference for all age ranges between those patients either above or below the 50th centile for poverty, clarifying the lack of relationship between age and socioeconomic status. A robust program of equal access to high-quality, well-coordinated cancer care, greater social support, and comprehensive interventions is required but may not be sufficient unless specific socioeconomic barriers that are unique to each group are effectively overcome to improve cancer-specific survival disparities between different racial groups and within each racial group based on socioeconomic determinant risk stratification.

An important and proven cost-effective interventional strategy would be to deploy a patient navigation program which refers to support and guidance offered to patients who come into contact with the health system with a presumptive diagnosis of cancer, either through screening or symptoms to ensure timely coordination and access to complex cancer therapies and to identify and remove socioeconomic barriers to care [41]. Patient navigation was originally conceived to address health disparities and patients’ risk for delays and loss to follow-up care among racial and ethnic minority and lower-income populations [42]. Such navigational interventions have been perceived by several groups of socially and economically disadvantaged individuals as beneficial and satisfactory, leading to better adherence to cancer treatments [41].

We anticipate our study will generate widespread interest among multidisciplinary teams to risk-stratify patients and facilitate efforts to address barriers in SEDH to achieve favorable cancer-specific survival outcomes.

## 5. Conclusions

This study showed the effects of SEDH, such as higher income levels, high school education, private insurance, and married marital status, have favorable survival in YoCRC patients. Some of these SEDH are also directly and indirectly related to the patients’ ethnicity. Most of the positive social factors are often interrelated. The presence of these factors should ultimately result in timely diagnosis and better CRC survival prognosis. CRC awareness, knowledge, and even utilization of medical services would differ with the education and health literacy. Further studies are recommended to assess the impact on cancer survival in YoCRC with specific targeted interventions to remove socioeconomic barriers among YoCRC patients.

## Figures and Tables

**Table 1:** A summary of demographic and clinicopathologic characteristics of YoCRC patients included in the study.

		Hispanic	Non-Hispanic American Indian	Non-Hispanic Asian Pacific Islander	Non-Hispanic Black	Non-Hispanic White	Total
		*n* = 2874	*n* = 164	*n* = 1676	*n* = 2305	*n* =10,126	*n* ** = 17,145**
**Age Range**	25–29 years	127	7	52	61	292	**539**
	30–34 years	288	1 4	113	141	717	**1273**
	35–39 years	487	28	217	294	1287	**2313**
	40–44 years	755	40	464	595	2702	**4556**
	45–49 years	1217	75	830	1214	5128	**8464**
**Sex**	Female	1371	77	846	1201	4754	**8249**
	Male	1503	87	830	1104	5372	**8896**
**Primary Site**	Appendix	95	8	37	57	304	**501**
	Ascending Colon	263	15	121	345	803	**1547**
	Cecum	252	9	115	348	922	**1646**
	Colon NOS	23	1	7	9	55	**95**
	Descending	182	8	126	179	526	**1021**
	Hepatic Flexure	73	5	52	64	209	**403**
	Overlapping	30	1	11	24	67	**133**
	Rectosigmoid	274	19	190	159	1118	**1760**
	Rectum	668	42	457	353	2807	**4327**
	Sigmoid	751	45	437	534	2569	**4336**
	Splenic Flexure	69	2	42	65	226	**404**
	Transverse Colon	194	9	81	168	520	**972**
**Histology**	Adenocarcinoma, NOS	1923	119	1224	1554	6839	**11,659**
	Adenocarcinoma In adenomatous polyp	198	11	105	186	994	**1494**
	Tubular Adenocarcinoma	1	0	0	1	4	**6**
	Serrated Adenocarcinoma	0	0	0	0	1	**1**
	Adenocarcinoma in adenomatous polyposis coli	11	0	3	4	17	**35**
	Adenocarcinoma in multiple Adenomatous polyps	3	0	2	2	2	**9**
	Adenocarcinoma in villous adenoma	79	1	25	54	213	**372**
	Villous Adenocarcinoma	1	0	1	2	2	**6**
	Adenocarcinoma in tubulovillous adenoma	267	17	154	248	1039	**1725**
	Clear cell Adenocarcinoma, NOS	1	0	0	0	2	**3**
	Cystadenocarcinoma, NOS	1	0	0	0	1	**2**
	Mucinous cystadenocarcinoma, NOS	2	0	1	2	8	**13**
	Mucinous adenocarcinoma	308	11	117	190	788	**1414**
	Mucin- producing adenocarcinoma	27	1	6	22	52	**108**
	Signet ring cell carcinoma	51	4	37	40	161	**293**
**Grade**	Well differentiated; Grade I	279	14	111	157	698	**1259**
	Moderately differentiated; Grade II	1901	110	1126	1592	6884	**11,613**
	Poorly differentiated; Grade Ill	420	23	287	316	1452	**2498**
	Undifferentiated; anaplastic; Grade IV	79	4	38	73	338	**532**
**Mucinous**	Non-Mucinous	2566	153	1559	2115	9338	**15,731**
	Mucinous	308	11	117	190	788	**1414**
**AJCC-7 TNM**	0	96	2	48	77	293	**516**
	I	469	30	276	385	1917	**3077**
	IIA	589	42	341	429	1922	**3323**
	IIB	70	1	28	49	212	**360**
	IIC	84	4	27	50	192	**357**
	IIIA	109	7	56	112	452	**736**
	IIIB	684	27	426	524	2409	**4070**
	IIIC	281	15	161	202	890	**1549**
	IVA	284	20	161	259	1058	**1782**
	IVB	184	14	1 43	204	702	**1247**
	IVC	24	2	9	14	79	**128**
**Procedure**	Colectomy or proctocolectomy resection in continuity	61	0	27	27	150	**265**
	Colectomy, NOS; Proctectomy, NOS	11	1	7	11	22	**52**
	Cryosurgery	0	0	0	0	1	**1**
	Electrocautery fulguration (hot forceps)	2	0	0	0	2	**4**
	Endoscopic polypectomy	42	1	32	36	144	**255**
	Excisional biopsy	16	2	9	14	104	**145**
	Hemicolectomy or subtotal colectomy	844	47	429	917	2769	**5006**
	Hemicolectomy or subtotal colectomy (and resection)	81	8	40	100	233	**462**
	Local tumor destruction, NOS	1	0	1	1	0	**3**
	Local tumor excision, electrocautery	11	2	4	5	35	**57**
	Local tumor excision, laser ablation	1	0	0	0	1	**2**
	Local tumor excision, laser excision	1	0	0	0	0	**1**
	Local tumor excision, NOS	13	1	4	8	32	**58**
	Partial colectomy (segmental resection)	1369	78	890	869	5145	**8351**
	Polypectomy, NOS	56	2	48	63	246	**415**
	Surgical polypectomy	6	1	1	7	16	**31**
	Total colectomy	209	12	107	122	679	**1129**
	Total colectomy (& resection of a contiguous organ)	11	1	6	8	52	**78**
	Total colectomy with ileorectal reconstruction	0	0	0	1	2	**3**
	Total colectomy with ileostomy, NOS	1	0	1	0	4	**6**
	Total colectomy with Pouch	1	0	0	0	1	**2**
	Total proctocolectomy (& resection of a contiguous organ)	3	0	1	5	10	**19**
	Total proctocolectomy with ileostomy (NOS)	1	1	0	2	6	**10**
	Total proctocolectomy with ileostomy & Pouch	3	0	0	0	7	**10**
	Total proctocolectomy, NOS	36	0	28	29	184	**277**
	Unknown if surgery performed; death certificate ON	26	3	8	17	56	**110**
	Wedge resection (segmental resection) & resection	68	4	33	63	225	**393**

**Table 2:** A summary of race specific survival controlled for each SEDH.

	Hispanic	Non-Hispanic American	Non-Hispanic Asian or Pacific Islander	Non-Hispanic Black	Non-Hispanic White
		Indian/Alaskan			
		Native			
Marital Status (Unmarried vs. Married)	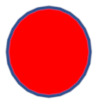	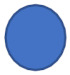	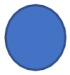	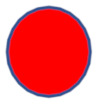	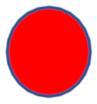
Immigration (Above vs. Below 50th centile)	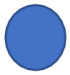	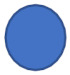	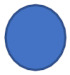	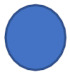	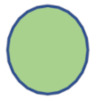
Employment Status (Above vs. Below 50th centile)	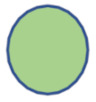	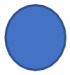	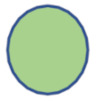	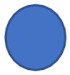	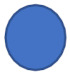
Poverty (Above vs. Below 50th centile)	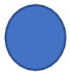	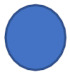	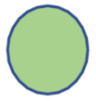	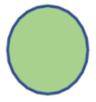	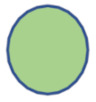
High School Education (Above vs. Below 50th centile)	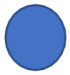	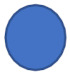	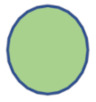	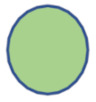	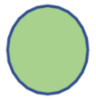
Insurance (Medicaid vs. Commercial)	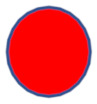	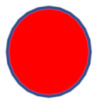	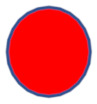	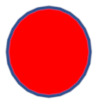	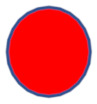
Household Income (Above vs. Below 50th centile)	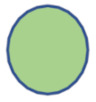	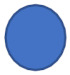	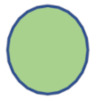	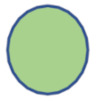	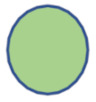

Red-adverse, Green-favorable, Blue-neutral.

**Table 3:** Univariate and multivariate results controlling for SEDH interactions.

Univariate Analysis				Multivariate Analysis			
Socioeconomic Determinant	HR	CI	*p*	Socioeconomic Determinant	HR	CI	*p*
Immigration	1.081	1.002–1.165	0.043	Immigration	1.011	0.927–1.102	0.808
Employment Status	0.89	0.826–0.96	0.002	Unemployment	1.006	0.91–1.112	0.809
Poverty	0.811	0.753–0.875	0.001	Poverty	0.887	0.787–0.999	**0.049**
High School Education	0.795	0.738–0.857	<0.0001	High School Education	0.875	0.791–0.967	**0.009**
Insurance (Commercial vs. Medicaid)	0.54	0.494–0.592	<0.0001	Insurance (Commercial vs. Medicaid)	0.597	0.542–0.659	**<0.0001**
Unmarried vs. Married	1.436	1.331–1.55	<0.0001	Unmarried vs. Married	1.305	1.201–1.419	**<0.0001**
Household Income	1.26	1.169–1.357	0.001	Household Income	1.136	0.994–1.1298	0.062

## Data Availability

American Community Survey datasets were accessed https://www.census.gov/data/developers/data-sets/acs-5year.2012.html (accessed on 3 March 2021).
